# The impact of respiratory specialist nurse-driven pulmonary rehabilitation care on respiratory function in ICU discharged severe pneumonia patients

**DOI:** 10.1097/MD.0000000000045887

**Published:** 2025-12-12

**Authors:** Qiaoyan Wu, Ying Wang, Aixia Zhu, Bing Ji, Yan Liang, Lan Zhu

**Affiliations:** a Department of Emergency and Critical Care Medicine, The Second Affiliated Hospital of Soochow University (The General Hospital of Nuclear Industry), Suzhou, Jiangsu, China.

**Keywords:** exercise capacity, ICU discharge, pulmonary rehabilitation care, respiratory function, respiratory specialist nurse, severe pneumonia

## Abstract

This study aims to evaluate the impact of respiratory specialist nurse-driven pulmonary rehabilitation care on the respiratory function of intensive care unit (ICU)-discharged severe pneumonia patients. This study is a retrospective cohort study, including 62 severe pneumonia patients discharged from the ICU of our hospital between February and December 2024. Propensity score matching was used to match the experimental group (received pulmonary rehabilitation care intervention) and the control group (received routine care), with 31 patients in each group. The study assessed the blood gas indicators, pulmonary function, dyspnea score, and exercise capacity of both groups on Day 1, Day 7, and at discharge. Mann–Whitney *U* test and Wilcoxon signed-rank test were used for between-group and within-group comparisons, with statistical significance set at *P* < .05. The baseline characteristics of the 2 groups were similar. The average age of the intervention group (n = 31) was 66 ± 7 years, with 67.7% male, while the average age of the control group (n = 31) was 65 ± 8 years, with 64.5% male. In the comparison between the intervention group and the control group, the intervention group showed significant improvement in respiratory function on Day 7 and at discharge. The arterial partial pressure of oxygen level in the intervention group (97.0 vs 88.0 mm Hg) and the arterial partial pressure of carbon dioxide level (40.0 vs 44.0 mm Hg) were significantly better than those of the control group (*P* < .05). Furthermore, the forced vital capacity (2.50 vs 2.20 L) and forced expiratory volume in 1 second (1.90 vs 1.65 L) in the intervention group were also significantly higher on Day 7 and at discharge compared to the control group (*P* < .05). The intervention group also showed significant improvement in the Modified Medical Research Council (Dyspnea Scale) score (2.0 vs 3.0) and the 6-minute walk distance (220 vs 180 m) (*P* < .05). Respiratory specialist nurse-driven pulmonary rehabilitation care significantly improved the oxygenation, pulmonary function, dyspnea, and exercise capacity of ICU-discharged severe pneumonia patients. This indicates that pulmonary rehabilitation care has a positive impact on the early recovery of severe pneumonia patients and is worth promoting in clinical practice.

## 1. Introduction

Severe pneumonia is a critical condition that arises after pneumonia, further complicated by respiratory failure or multiple organ dysfunction. The pathogenesis typically involves severe septicemia and bacteremia caused by infections from various pathogens. Patients often present with severe symptoms such as shock, rapid blood pressure drop, altered consciousness, delirium, and agitation, which can be life-threatening and are associated with a high mortality rate. In recent years, the incidence of severe pneumonia has shown a continuous upward trend, becoming a key research area in critical care medicine and respiratory medicine, especially in ICUs (intensive care units), where it is one of the most common respiratory diseases.^[1,2]^ To improve patient clinical outcomes, mechanical ventilation, as an important treatment for severe pneumonia, can effectively improve oxygenation status and promote recovery.^[3]^ However, prolonged mechanical ventilation, sedation, and bed rest can lead to muscle atrophy, impaired respiratory muscle function, and other issues, all of which are risk factors for ICU-acquired weakness syndrome, atelectasis, aspiration pneumonia, and other complications.^[4]^

Pulmonary rehabilitation, as an adjunctive measure in the treatment of severe pneumonia patients, has been widely applied in clinical practice to control and alleviate symptoms, reduce functional disabilities, and improve quality of life.^[5]^ In recent years, an increasing number of studies have pointed out that severe pneumonia patients need pulmonary rehabilitation to promote functional recovery during their treatment, especially during the transitional care period after patients are transferred to general wards. Research has shown that inadequate transitional care after ICU discharge may lead to patient relapse, prolonged hospital stays, or readmission to the ICU.^[6]^ Therefore, strengthening transitional care after ICU discharge and ensuring continuous and high-quality care interventions are key to improving patient outcomes.

The concept of “continuity of care” was first proposed by Meleis et al in 1986, emphasizing the need to ensure continuity and therapeutic effectiveness when patients transition from one care setting to another.^[7]^ In recent years, continuity of care has been widely applied in the transitional care of ICU patients, especially abroad, where Critical Care Outreach Teams have become a standard setting in many hospitals, aimed at ensuring that patients discharged from the ICU receive timely interventions and high-quality care.^[8,9]^ Respiratory specialist nurses play a crucial role in this model by providing specialized care and support during the transition from ICU to general wards, as demonstrated in recent international studies.^[10]^ In China, the transitional care model for ICU patients has started later, and although some scholars have explored and implemented transitional care models, such as Xu Shuhua et al in 2013, who used this model for intervention in patients with severe brain injury, it was found to effectively reduce adverse nursing events and the risk of readmission to the ICU.^[10]^ However, there are still issues in the transitional care of ICU-discharged patients, such as a shortage of nursing staff and inconsistent nursing content.^[11,12]^ Recently, emerging evidence from both domestic and international sources has highlighted the importance of respiratory nurse-led interventions in improving post-ICU recovery, particularly in the management of respiratory function and prevention of complications.

This study focuses on the pulmonary rehabilitation care for ICU-discharged severe pneumonia patients and aims to explore the impact of respiratory specialist nurse-led pulmonary rehabilitation interventions on patient respiratory function. Respiratory specialist nurses, as professionals who have received specialized training and obtained relevant certification, possess a higher level of theoretical and practical skills and can play a key role in managing respiratory diseases, improving quality of life, and reducing disease deterioration.^[13,14]^ However, research in this field is still limited in China, especially regarding respiratory specialist nurse-led rehabilitation interventions for severe pneumonia patients after ICU discharge, which have not been fully applied or validated. Therefore, this study aims to evaluate the application effects of respiratory specialist nurse-led pulmonary rehabilitation care in ICU-discharged severe pneumonia patients, providing new clinical evidence to improve respiratory function, shorten hospital stays, and reduce complications.

## 2. Materials and methods

### 2.1. Study design

This study was approved by the ethics committee of the Second Affiliated Hospital of Soochow University (The General Hospital of Nuclear Industry). This study is a retrospective cohort study aimed at evaluating the impact of respiratory specialist nurse-led pulmonary rehabilitation care on the respiratory function of ICU-discharged severe pneumonia patients. The study subjects were 62 severe pneumonia patients discharged from the ICU between February and December 2024 at our hospital. By reviewing patients’ hospitalization records, they were divided into an experimental group, which received pulmonary rehabilitation care intervention, and a control group, which did not receive any intervention. Propensity score matching (PSM) was used to control potential confounding factors between the groups, ultimately matching 31 patients in each group based on confounding factors.

*Inclusion criteria*: Age ≥ 18 years; primary diagnosis meets the diagnostic criteria for severe pneumonia as defined in the “Community-Acquired Pneumonia Traditional Chinese Medicine Diagnosis and Treatment Guidelines (2018 Revised Edition),” with typical clinical manifestations and imaging examination results; ICU discharge patients, meeting the ICU discharge criteria of our hospital, and transferred to our department for continued treatment after ICU care; patients who have been weaned from mechanical ventilation or have successfully recovered from ventilator treatment and are capable of independent breathing; patients with clear consciousness, no severe consciousness impairment, and able to communicate effectively and cooperate with treatment.

*Exclusion criteria*: Patients with severe heart, brain, or kidney dysfunction that affect the recovery of respiratory function; patients with advanced malignant tumors, poor expected treatment outcomes, and a short life expectancy; patients with severe mental disorders or cognitive impairments, unable to understand or follow rehabilitation training; patients with severe neuromuscular injuries or other neuromuscular diseases affecting respiratory function recovery; patients who experienced massive bleeding, bleeding risks, or were highly suspected of having pulmonary embolism (including confirmed pulmonary embolism); patients or family members who refused treatment, or patients who voluntarily discharged themselves and did not continue treatment.

### 2.2. Research methods

#### 2.2.1. Control group

Patients in the control group received routine pulmonary rehabilitation nursing measures. Under the guidance of physicians, appropriate oxygen therapy was administered to ensure adequate blood oxygen saturation, including low- or high-flow oxygen therapy as needed. Nursing staff assisted patients with repositioning and back percussion to promote sputum drainage and maintain airway patency. Patients were also guided to perform effective coughing and expectoration exercises to help clear respiratory secretions. Deep breathing training was encouraged to improve lung ventilation, and patients were instructed to engage in appropriate in-bed movements and progressive out-of-bed activities to gradually restore lung function.

#### 2.2.2. Intervention group

Patients in the intervention group received a customized pulmonary rehabilitation care program, which involved comprehensive interventions from multiple aspects, including the establishment of a pulmonary rehabilitation nursing team, development of individualized care plans, and implementation of continuous supervision and quality control.

##### 2.2.2.1. Establishment of the pulmonary care team and role allocation

A pulmonary rehabilitation nursing team was established by the respiratory specialist nurse within the department. The team consisted of ICU physicians, the head nurse, the respiratory specialist nurse, and 2 charge nurses. The ICU physician was responsible for the initial assessment of the patient’s condition and pulmonary status. The head nurse led the team, coordinated staffing, formulated work plans, and supervised the execution of the nursing processes. The respiratory specialist nurse was in charge of training team members, implementing the care program, and performing quality control to ensure that each nursing intervention was properly carried out. The team also included experienced and responsible charge nurses, who were responsible for implementing specific nursing tasks and collecting relevant data.

##### 2.2.2.2. Development of the care plan

To formulate a pulmonary rehabilitation care plan suitable for patients in this hospital, team members conducted an extensive review of relevant domestic and international literature, referencing the Guidelines for the Diagnosis and Treatment of Community-Acquired Pneumonia in Traditional Chinese Medicine as well as other related guidelines, publications, and expert consensuses. Based on research findings and expert opinions, a comprehensive rehabilitation plan was developed, covering exercise types, duration, frequency, and intensity. The plan was finalized after expert panel reviews and 2 rounds of Delphi consultations, and its feasibility was verified through a pilot test to ensure practicality and effectiveness.

##### 2.2.2.3. Team member training and assessment

All team members underwent a 4-week unified training and assessment program. The training included fundamental knowledge of pulmonary rehabilitation nursing, detailed implementation protocols, and key precautions. Each week featured a different training module:

Week 1: Basic concepts of pulmonary rehabilitation.

Week 2: Detailed explanation of the care plan and execution standards.

Week 3: On-site simulation drills and Q&A.

Week 4: Case-based simulation assessments.

Members who failed the assessment underwent retraining until they fully mastered the plan.

##### 2.2.2.4. Implementation of the pulmonary rehabilitation care plan

*Assisted breathing*: According to the patient’s respiratory status and oxygen saturation levels, appropriate assisted breathing support was provided, including thoracic relaxation techniques, low-flow oxygen therapy, and high-flow oxygen therapy to ensure adequate ventilation and oxygen delivery.

*Airway management*: ICU physicians assessed patients’ pulmonary status, and nursing team members used an airway grading scale for evaluation. Based on the score, interventions included nebulized inhalation, chest physiotherapy, and assisted coughing techniques. Cough techniques included controlled coughing, triple coughing, Heimlich maneuver, and anterior chest compression methods to aid in sputum clearance.

*Breathing training*: Individualized breathing exercises were designed based on patients’ physiological conditions. These included diaphragmatic breathing, pursed-lip breathing, and respiratory muscle training. Each exercise was adjusted in frequency and duration according to the patient’s physical tolerance to ensure both effectiveness and safety.

Exercise training: Exercise programs were tailored based on patients’ muscle strength and vital signs, including passive and active limb movements, resistance band training, and aerobic exercises. Intensity and frequency were gradually increased to enhance physical strength and support functional recovery.

*Training intensity control*: The intensity and frequency of each training session were adjusted based on continuous monitoring of vital signs and dyspnea assessments. This ensured that the training did not impose additional burden on the patient or trigger other complications.

##### 2.2.2.5. Quality control

During implementation, the head nurse was responsible for supervising the execution of the care plan and recording daily nursing activities. Team members used an instant messaging platform to compile and report issues encountered during care delivery, enabling timely adjustments and improvements to the interventions. Quality control measures included regular evaluation of patient recovery status, effectiveness of care, and feasibility of the plan. Continuous optimization of the care plan was guided by patient feedback and clinical outcomes.

### 2.3. Data collection

The following clinical data and assessment indicators were extracted from patient medical records and nursing documentation:

#### 2.3.1. Arterial blood gas indicators

On the 1st and 7th days after ICU discharge, the oxygen delivery method and inspired oxygen concentration (FiO_2_) were recorded. A professional nurse drew 2 mL of radial arterial blood for immediate blood gas analysis to measure arterial partial pressure of oxygen (PaO_2_) and arterial partial pressure of carbon dioxide (PaCO_2_). The ratio of arterial oxygen to inspired oxygen (oxygenation index) (PaO_2_/FiO_2)_ was calculated to evaluate changes in oxygenation and ventilation status.

#### 2.3.2. Pulmonary function indicators

On the 1st day, 7th day after ICU discharge, and at discharge, patients underwent pulmonary function tests assisted by a specialized respiratory therapist. Forced vital capacity (FVC) and forced expiratory volume in 1 second (FEV_1_) were measured, and the FEV_1_/FVC percentage was calculated to assess recovery of pulmonary ventilation. If the patient’s physical condition prevented completion of the full test, the maximum tolerable measurements were recorded.

#### 2.3.3. Dyspnea score

On the 1st day, 7th day after ICU discharge, and at discharge, the severity of subjective dyspnea was assessed using the Modified Medical Research Council (mMRC) Dyspnea Scale. Trained nursing staff performed the scoring, based on the patient’s subjective perception during minimal exertion.

#### 2.3.4. Exercise capacity assessment

On the 1st day, 7th day after ICU discharge, and at discharge, the 6-minute walk distance (6MWD) test was conducted following the 2002 American Thoracic Society guidelines for the 6-minute walk test. The test was supervised by healthcare professionals, and the total distance walked at the patient’s maximum tolerable speed was recorded as an indicator of physical endurance and recovery level.

### 2.4. Statistical analysis

Data analysis in this study was performed using SPSS version 22.0 (IBM Corporation, Armonk). The Shapiro–Wilk test was used to assess the normality of all continuous variables, and most data did not conform to a normal distribution. Therefore, all continuous variables were expressed as median (interquartile range), and differences between the 2 groups were compared using the Mann–Whitney *U* test. Categorical variables were expressed as frequencies and percentages, and the Chi-square test was used to compare group differences. To control for potential confounding variables, this study employed the PSM method. Propensity scores were calculated using a logistic regression model that included baseline demographic characteristics (age, gender, body mass index, and smoking history) and disease severity indicators (Acute Physiology and Chronic Health Evaluation II score, Sequential Organ Failure Assessment score, duration of mechanical ventilation, and ICU length of stay). A 1:1 nearest-neighbor matching without replacement was performed, with a caliper width set at 0.2 of the standard deviation of the logit of the propensity score to reduce selection bias. After matching, each group included 31 patients. There were no statistically significant differences in baseline demographic characteristics or disease severity indicators between the 2 groups (*P* > .05), providing a reliable foundation for evaluating the intervention outcomes. Changes in arterial blood gas parameters, pulmonary function, dyspnea scores, and exercise capacity were evaluated on the 1st day, 7th day after ICU discharge, and at hospital discharge. Since all data represented repeated measurements, the Wilcoxon signed-rank test was used to assess within-group changes, while the Mann–Whitney *U* test was used to compare between-group differences. Considering that repeated comparisons were conducted at 3 time points (Day 1, Day 7, and discharge), the Bonferroni correction was applied to adjust for multiple testing, with the threshold for statistical significance set at *P* < .017.

## 3. Results

### 3.1. Baseline characteristics after propensity score matching

Analysis of baseline characteristics showed that, after propensity score matching, the 2 groups were comparable and balanced (*P* > .05). There were no significant differences between the intervention group (n = 31) and the control group (n = 31) in demographic characteristics such as age (66 ± 7 vs 65 ± 8 years), gender (67.7% vs 64.5% male), and body mass index (25.0 ± 3.2 vs 24.5 ± 3.0 kg/m²) (*P* = .73; *P* = .758; *P* = .591), as shown in Table 1.

**Table 1:** Baseline demographic and clinical characteristics of the control and intervention groups.

Variable	Control group (n = 31)	Intervention group (n = 31)	Statistic	*P*-value
Age (yr)	65 ± 8	66 ± 7	*t* = −0.347	.73
Gender (male, n, %)	20 (64.5%)/11 (35.5%)	21 (67.7%)/10 (32.3%)	*χ*² = 0.095	.758
BMI (kg/m²)	24.5 ± 3.0	25.0 ± 3.2	*t* = −0.539	.591
Smoking history (n, %)	15 (48.4%)	16 (51.6%)	*χ*² = 0.040	.842
APACHE II score	22.0 ± 6.0	21.0 ± 5.5	*t* = 0.458	.649
SOFA score	7.5 ± 2.3	7.8 ± 2.0	*t* = −0.407	.685
Mechanical ventilation duration (d)	8.0 ± 3.2	7.2 ± 2.8	*t* = 1.134	.26
ICU stay duration (d)	10.0 ± 4.0	9.0 ± 3.5	*t* = 1.074	.288
Hypertension (n, %)	12 (38.7%)	14 (45.2%)	*χ*² = 0.233	.63
Diabetes (n, %)	10 (32.3%)	12 (38.7%)	*χ*² = 0.276	.599
COPD (n, %)	5 (16.1%)	6 (19.4%)	*χ*² = 0.142	.707
Coronary artery disease (n, %)	4 (12.9%)	5 (16.1%)	*χ*² = 0.128	.721
PaO_2_/FiO_2_ ratio at Transfer (mm Hg)	300 (270, 330)	310 (280, 340)	*U* = 436.5	.474
FVC at transfer (L)	2.00 (1.85, 2.20)	2.10 (1.90, 2.30)	*U* = 437.0	.481
FEV_1_ at transfer (L)	1.50 (1.30, 1.60)	1.55 (1.40, 1.65)	*U* = 454.0	.617

APACHE II = Acute Physiology and Chronic Health Evaluation II, BMI = body mass index, FVC = forced vital capacity, FEV1 = forced expiratory volume in 1 second, ICU = intensive care unit, PaO2/FiO2 = ratio of arterial oxygen to inspired oxygen (oxygenation index).

Regarding disease severity, the 2 groups were well matched in Acute Physiology and Chronic Health Evaluation II scores (21.0 ± 5.5 vs 22.0 ± 6.0) and sequential organ failure assessment scores (7.8 ± 2.0 vs 7.5 ± 2.3) (*P* > .05). Although slight differences were observed in mechanical ventilation duration (7.2 ± 2.8 vs 8.0 ± 3.2 days) and ICU length of stay (9.0 ± 3.5 vs 10.0 ± 4.0 days), these were not statistically significant (*P* > .05).

Respiratory function indicators, including PaO_2_/FiO_2_ ratio, FVC, and FEV_1_, were also balanced between groups (*P* > .05). The distribution of comorbidities showed no significant differences either (*P* > .05). This good baseline balance provided a reliable foundation for evaluating the subsequent effects of the intervention.

### 3.2. Comparative analysis of arterial blood gas indicators before and after intervention

In the comparison of arterial blood gas changes before and after pulmonary rehabilitation care between the 2 groups, there were no statistically significant differences in any parameters on Day 1 after ICU discharge (*P* > .05), indicating that the initial respiratory function status was comparable between groups (see Table 2). By Day 7, however, the intervention group demonstrated significant improvements. The PaO_2_ level in the intervention group reached 97.0 (90.0, 105.0) mm Hg, notably higher than the control group’s 88.0 (80.0, 95.0) mm Hg, with a statistically significant difference (*U* = 336.5, *P* = .012). Similarly, the PaCO_2_ level was significantly lower in the intervention group at 40.0 (37.0, 44.0) mm Hg, compared to 44.0 (41.0, 48.0) mm Hg in the control group (*U* = 338.0, *P* = .014), reflecting improved ventilation function. Furthermore, the PaO_2_/FiO_2_ ratio in the intervention group was markedly higher at 360 (330, 390), in contrast to 320 (290, 350) in the control group, with the difference being statistically significant (*U* = 318.0, *P* = .008). These results indicate that respiratory specialist nurse-led pulmonary rehabilitation care plays a positive role in enhancing oxygenation and carbon dioxide clearance in severe pneumonia patients after ICU discharge.

**Table 2:** Comparison of blood gas indicators between the intervention group and the control group on Day 1 and Day 7.

Variable	Control group (n = 31)	Intervention group (n = 31)	Statistic	*P*-value
Day 1 PaO_2_ (mm Hg, $\bar{x}\pm s$)	82.0 (75.0, 91.0)	83.0 (76.0, 90.0)	*U* = 464.0	.653
Day 7 PaO_2_ (mm Hg, $\bar{x}\pm s$)	88.0 (80.0, 95.0)	97.0 (90.0, 105.0)	*U* = 336.5	.012
Day 1 PaCO_2_ (mm Hg, $\bar{x}\pm s$)	45.0 (41.0, 50.0)	45.0 (42.0, 49.0)	*U* = 472.5	.702
Day 7 PaCO_2_ (mm Hg, $\bar{x}\pm s$)	44.0 (41.0, 48.0)	40.0 (37.0, 44.0)	*U* = 338.0	.014
Day 1 PaO_2_/FiO_2_	300 (270, 330)	310 (280, 340)	*U* = 436.5	.474
Day 7 PaO_2_/FiO_2_	320 (290, 350)	360 (330, 390)	*U* = 318.0	.008

PaCO2 = arterial partial pressure of carbon dioxide, PaO_2_ = arterial partial pressure of oxygen.

### 3.3. Comparison of pulmonary function indicators

In this study, there were no significant differences in pulmonary function indicators (FVC, FEV_1_, FEV_1_/FVC) between the intervention and control groups on Day 1 after ICU discharge, indicating similar baseline pulmonary function status (see Table 3). Specifically, both FVC and FEV_1_ values were comparable between the 2 groups on Day 1, with no statistically significant differences (*P* > .05). However, by Day 7 and at discharge, the intervention group demonstrated significantly better pulmonary function outcomes than the control group. Notably, on Day 7, the FVC and FEV_1_ values in the intervention group were significantly higher than those in the control group (*P* < .05), highlighting the positive effect of pulmonary rehabilitation nursing on lung function recovery. This trend continued at discharge, with the intervention group maintaining significantly higher FVC and FEV_1_ values, further underscoring their advantage in pulmonary recovery. Although the FEV_1_/FVC ratio did not show a statistically significant difference between the 2 groups (*P* > .05), the intervention group consistently showed more pronounced improvements in all pulmonary function indicators across all time points. In summary, pulmonary rehabilitation care significantly promoted lung function recovery in the intervention group, with particularly notable improvements observed on Day 7 and at discharge.

**Table 3:** Comparison of pulmonary function parameters between the intervention group and the control group at Day 1, Day 7, and discharge.

Variable	Time point	Control group (n = 31)	Intervention group (n = 31)	*U* statistic	*P*-value
FVC (L)	Day 1	2.00 (1.85, 2.20)	2.10 (1.90, 2.30)	*U* = 437.0	.481
	Day 7	2.20 (2.00, 2.45)	2.50 (2.30, 2.80)	*U* = 310.0	.009
	Discharge	2.40 (2.10, 2.60)	2.80 (2.50, 3.00)	*U* = 298.5	.006
FEV_1_ (L)	Day 1	1.50 (1.30, 1.60)	1.55 (1.40, 1.65)	*U* = 454.0	.617
	Day 7	1.65 (1.45, 1.80)	1.90 (1.70, 2.00)	*U* = 312.0	.011
	Discharge	1.80 (1.60, 2.00)	2.10 (1.90, 2.30)	*U* = 293.0	.004
FEV_1_/FVC (%)	Day 1	75.0 (70.0, 78.0)	74.0 (71.0, 77.0)	*U* = 470.0	.725
	Day 7	75.5 (72.0, 78.0)	76.0 (73.0, 79.0)	*U* = 430.0	.417
	Discharge	76.0 (72.0, 79.0)	77.0 (74.0, 80.0)	*U* = 419.5	.361

FVC = forced vital capacity, FEV1 = forced expiratory volume in 1 second.

### 3.4. Comparison of Dyspnea scores

In this study, dyspnea severity was assessed using the mMRC scale on Day 1, Day 7 after ICU discharge, and at hospital discharge for both the intervention and control groups. As shown in Table 4, there was no significant difference in mMRC scores between the 2 groups on Day 1, indicating comparable initial levels of dyspnea symptoms. Specifically, both groups had a median mMRC score of 3.5 (3.0, 4.0), with no statistical significance observed (*U* = 465.0, *P* = .973).

**Table 4:** Comparison of mMRC Dyspnea scores between the intervention group and the control group at Day 1, Day 7, and discharge.

Variable	Time point	Control group (n = 31)	Intervention group (n = 31)	*U* statistic	*P*-value
mMRC score	Day 1	3.5 (3.0, 4.0)	3.5 (3.0, 4.0)	*U* = 465.0	.973
	Day 7	3.0 (2.0, 3.5)	2.0 (1.5, 2.5)	*U* = 314.5	.003
	Discharge	2.5 (2.0, 3.0)	1.5 (1.0, 2.0)	*U* = 290.0	.001

mMRC = Modified Medical Research Council (Dyspnea Scale).

However, by Day 7 and at discharge, the intervention group demonstrated significantly lower mMRC scores compared to the control group, indicating a notable improvement in perceived breathlessness. On Day 7, the intervention group’s median score dropped to 2.0 (1.5, 2.5), significantly lower than the control group’s 3.0 (2.0, 3.5), with a statistically significant difference (*U* = 314.5, *P* = .003). By the time of discharge, the intervention group showed further improvement, with a score of 1.5 (1.0, 2.0), compared to 2.5 (2.0, 3.0) in the control group (*U* = 290.0, *P* = .001). These results suggest that respiratory specialist nurse-led pulmonary rehabilitation care effectively alleviated dyspnea symptoms over time, with significant improvements evident as early as Day 7 and sustained through discharge.

### 3.5. Comparison of exercise capacity

To assess physical recovery, the 6MWD test was used to compare the exercise capacity of the 2 groups on Day 1, Day 7 after ICU discharge, and at hospital discharge (see Table 5). On Day 1, there was no statistically significant difference between the intervention group and the control group in walking distance (160 [140, 180] m versus 150 [130, 170] m, respectively [*U* = 451.0, *P* = .453]) indicating comparable baseline physical capacity. However, as rehabilitation interventions progressed, the intervention group showed significantly better improvement in exercise capacity on both Day 7 and at discharge. On Day 7, the median 6MWD in the intervention group was 220 (200, 240) m, significantly greater than the control group’s 180 (160, 200) m (*U* = 315.0, *P* = .002). By discharge, the intervention group further improved to 250 (230, 270) m, compared to 200 (180, 220) m in the control group, with the difference remaining statistically significant (*U* = 290.0, *P* = .001). These findings clearly demonstrate that respiratory specialist nurse-led pulmonary rehabilitation care effectively enhanced patients’ physical endurance and functional recovery following ICU discharge.

**Table 5:** Comparison of six-minute walk distance between the intervention group and the control group at Day 1, Day 7, and discharge.

Variable	Time point	Control group (n = 31)	Intervention group (n = 31)	*U* statistic	*P*-value
6-minute walk distance (m)	Day 1	150 (130, 170)	160 (140, 180)	*U* = 451.0	.453
	Day 7	180 (160, 200)	220 (200, 240)	*U* = 315.0	.002
	Discharge	200 (180, 220)	250 (230, 270)	*U* = 290.0	.001

## 4. Discussion

This study aimed to investigate the effects of respiratory specialist nurse-led pulmonary rehabilitation care on respiratory function in ICU-discharged patients with severe pneumonia. The results demonstrated that this intervention model offers significant advantages in improving oxygenation, ventilation, pulmonary function, perceived dyspnea, and physical capacity. Following propensity score matching, the intervention and control groups were comparable in demographic and disease severity characteristics, lending reliability and representativeness to the study findings.

By Day 7 post-ICU discharge, the intervention group showed significantly higher PaO_2_ levels, lower PaCO_2_ levels, and improved PaO_2_/FiO_2_ ratios compared to the control group, indicating more rapid recovery of oxygenation and ventilation function. The likely mechanism behind this improvement lies in the effectiveness of the pulmonary rehabilitation program led by respiratory specialist nurses, which integrates targeted breathing training, airway management, and ventilatory support. Techniques such as diaphragmatic and pursed-lip breathing enhance the coordination of the diaphragm and accessory respiratory muscles, thereby improving alveolar ventilation.^[15]^ Meanwhile, interventions like nebulized inhalation, chest percussion, and assisted coughing help clear airway secretions, reduce the risk of atelectasis and secondary infections, and improve lung compliance and oxygenation.^[16,17]^

In terms of pulmonary function, the intervention group demonstrated superior outcomes in FVC and FEV_1_ on both Day 7 and at discharge, likely due to the early, structured implementation of respiratory and physical training. Based on individualized assessments, respiratory specialist nurses designed tailored rehabilitation plans that controlled training intensity and frequency, promoting recovery of lung volumes under safe conditions.^[18]^ Moreover, aerobic exercises and resistance band training played crucial roles in enhancing respiratory muscle endurance and preventing disuse muscle atrophy. These interventions helped patients break the vicious cycle of “prolonged bed rest – muscle weakness – poor lung function – continued immobility,” thereby facilitating meaningful improvements in pulmonary function.^[19]^

Although the intervention group demonstrated significant improvements in FEV_1_ and FVC compared to the control group, the FEV_1_/FVC ratio did not differ significantly between the 2 groups. A possible explanation is that both FEV_1_ and FVC increased in parallel during rehabilitation, leading to proportional changes and thus relatively stable ratios. The FEV_1_/FVC ratio is typically more sensitive in distinguishing obstructive ventilatory defects, whereas our patient population primarily exhibited restrictive changes after severe pneumonia and prolonged ICU stay. Therefore, improvements were more apparent in absolute lung volumes (FEV_1_, FVC) rather than in the ratio.

Subjective dyspnea, as measured by the mMRC score, significantly decreased in the intervention group, indicating a noticeable reduction in patients’ perception of breathlessness. The improvement in dyspnea may be closely related to the objective recovery of pulmonary function, as well as to the psychological support, anxiety reduction, and breathing pattern training provided by the nursing team.^[20]^ The professional guidance and continuous monitoring by respiratory specialist nurses enhanced patients’ ability to manage their symptoms and improved their rehabilitation adherence.

In terms of exercise capacity, the intervention group showed significantly better 6-minute walk distances on both Day 7 and at discharge, suggesting faster physical recovery. The exercise training in pulmonary rehabilitation promotes skeletal muscle oxygenation and enhances cardiopulmonary endurance, thereby improving activity tolerance. This type of intervention not only improves patients’ exercise capacity but also indirectly reflects improvements in their overall physical condition. Particularly in the context of ICU-acquired weakness syndrome, respiratory specialist nurse-led rehabilitation training plays a critical role in early functional recovery.^[21]^

In conclusion, this study confirms that respiratory specialist nurse-led pulmonary rehabilitation care significantly improves the respiratory function and overall recovery of ICU-discharged severe pneumonia patients through multidimensional interventions. The advantages of this model lie in its precise assessments, professional interventions, standardized processes, and strong continuity, addressing the shortcomings of routine nursing care in terms of specialty depth and systematization. This model enhances patient discharge outcomes and holds strong clinical promotion potential.

Although the results of this study are promising, there are certain limitations. First, this study is a single-center, retrospective study with a limited sample size, which may lead to selection bias and information bias. The relatively small number of patients (n = 62) reflects all eligible severe pneumonia cases discharged from the ICU in our hospital during the study period. To minimize potential confounding effects despite the small sample, we applied PSM based on key demographic and clinical variables, which ensured baseline comparability between groups and enhanced the internal validity of the results. Nevertheless, future studies should expand the sample size and perform multicenter validation to further improve the robustness and generalizability of the findings. Second, since some indicators rely on patient subjective cooperation, such as pulmonary function tests and mMRC scores, they may be influenced by patients’ understanding and physical limitations. It is recommended that future research incorporate more objective measures, such as respiratory muscle electromyography and lung ultrasound assessments. Additionally, the intervention period in this study was relatively short, and the long-term effects, such as readmission rates and long-term pulmonary function recovery, were not evaluated. Future studies should extend the follow-up period for continuous observation.

In summary, respiratory specialist nurse-led pulmonary rehabilitation care significantly improves oxygenation, pulmonary function, subjective dyspnea, and exercise capacity in ICU-discharged severe pneumonia patients, highlighting its value in post-ICU continuity of care. It is recommended to promote this type of nursing intervention in clinical practice and further explore its application effects and mechanisms in patients with various types of respiratory diseases.

## Author contributions

**Conceptualization:** Qiaoyan Wu, Lan Zhu.

**Data curation:** Ying Wang, Lan Zhu.

**Formal analysis:** Lan Zhu.

**Investigation:** Aixia Zhu, Lan Zhu.

**Methodology:** Aixia Zhu, Lan Zhu.

**Supervision:** Yan Liang, Lan Zhu.

**Validation:** Bing Ji, Lan Zhu.

**Visualization:** Bing Ji, Lan Zhu.

**Writing – original draft:** Qiaoyan Wu, Lan Zhu.

**Writing – review & editing:** Qiaoyan Wu, Lan Zhu.
